# Telemonitoring in Heart Failure: A Review of Monitoring Techniques and Clinical Outcomes

**DOI:** 10.7759/cureus.102215

**Published:** 2026-01-24

**Authors:** Sara Szukalska, Marta Karczewska, Kamil Wróblewski, Karolina Lichwala, Lukasz Siwek, Angelika Samborska, Barbara Balajewicz, Paulina Wróblewska

**Affiliations:** 1 General Medicine, Medical University of Silesia, Katowice, POL; 2 General Practice, Dr. Tytus Chałubiński Specialist Hospital in Radom, Radom, POL; 3 General Practice, Karol Marcinkowski Medical University in Poznań, Poznan, POL; 4 Medicine, Szpital Miejski w Zabrzu, Zabrze, POL

**Keywords:** clinical cardiology, digital healthcare, heart failure, remote monitoring, telemedicine, telemonitoring

## Abstract

Heart failure (HF) remains a significant cause of global morbidity and mortality, as well as a major contributor to healthcare expenditure, primarily due to frequent disease exacerbations and high rates of hospital readmissions. The increasing burden of chronic HF, alongside the limitations of traditional outpatient follow-up, has intensified interest in telemonitoring as a means of supporting the early detection of clinical deterioration and optimising long-term management. This narrative review evaluates the current evidence on telemonitoring for HF, focusing particularly on available technologies, clinical effectiveness, implementation barriers and priorities for future research. Open-access literature indexed in PubMed was reviewed, including randomised controlled trials, systematic reviews and meta-analyses involving adult patients with HF. Both invasive and non-invasive monitoring approaches, as well as mobile-based solutions and integrated, multidisciplinary care models, were considered. Overall, most studies suggest that telemonitoring interventions are linked to fewer HF-related hospitalisations, particularly when combined with structured clinical feedback and prompt therapeutic adjustments. However, evidence for an effect on all-cause mortality remains inconsistent. More favourable outcomes are observed in programmes that include an active clinical response, rather than passive data collection alone. Substantial variability in study design, patient populations, monitoring intensity and outcome measures contributes to the inconsistency in reported effectiveness. Although emerging technologies such as mobile health applications and implantable monitoring devices show promise, their implementation in the real world is often hindered by issues relating to patient adherence, costs, digital literacy and the absence of standardised protocols. Telemonitoring is a valuable addition to HF management, particularly for reducing hospital admissions and enabling early intervention. Its long-term effectiveness hinges on selecting the right patients, choosing the right monitoring method, and integrating it into multidisciplinary care pathways. Future research should prioritise protocol standardisation, cost-effectiveness evaluation, and the inclusion of underrepresented patient groups, in order to better define the role of telemonitoring in routine HF care.

## Introduction and background

Heart failure (HF) is one of the most prevalent chronic conditions worldwide and a major driver of healthcare expenditure, largely due to frequent hospitalisations and the long-term nature of care it requires. Despite significant advances in pharmacological and device-based therapies, HF remains characterised by clinical instability and a progressive decline in quality of life, including reduced functional capacity and increased symptom burden [1]. HF-related hospital admissions contribute disproportionately to total healthcare costs and are strongly associated with poor clinical outcomes. The burden of HF is further exacerbated by population ageing and the rising prevalence of multimorbidity, which complicate disease management and follow-up [2,3]. Traditional care models based on scheduled outpatient visits may not adequately capture early signs of decompensation, particularly in high-risk patients. Consequently, there is an increasing need for alternative care approaches that enable continuous or near-continuous monitoring and facilitate timely clinical intervention.

HF is a heterogeneous syndrome comprising several distinct phenotypes, of which heart failure with reduced ejection fraction (HFrEF) and heart failure with preserved ejection fraction (HFpEF) are the most common. These phenotypes differ in their underlying pathophysiology, clinical manifestations, and response to therapy, which may influence the effectiveness of telemonitoring interventions. Patients with HFpEF are typically older and have a higher burden of comorbidities, such as hypertension, diabetes and atrial fibrillation, which may limit the utility of monitoring strategies focused primarily on haemodynamic or volume-related parameters. In contrast, patients with HFrEF and a predominantly congestive profile may derive greater benefit from telemonitoring approaches that enable early detection of fluid accumulation and timely adjustment of therapy.

Early detection of clinical deterioration is a key component of HF management, as timely intervention may prevent progression to overt decompensation and reduce the risk of hospitalisation. Symptoms such as worsening dyspnoea, peripheral oedema or rapid weight gain often emerge days or even weeks before an acute exacerbation; however, they are frequently identified too late to permit effective outpatient intervention [4,5]. This delay reflects both the gradual progression of symptoms and the reliance on patient self-reporting, which may be inaccurate or inconsistent. Standard outpatient care models based on scheduled clinic visits are often unable to capture these dynamic changes in clinical status, particularly during high-risk periods such as the early post-discharge phase [3,6]. Following hospitalisation for acute HF, patients remain vulnerable to recurrent decompensation, yet monitoring intensity typically decreases after discharge. This gap in surveillance underscores the need for approaches that enable more continuous assessment of physiological trends and symptom fluctuations.

Telemonitoring offers a potential means of shifting clinical assessment from episodic, clinic-based encounters to the patient’s home environment. By enabling regular or continuous collection of physiological parameters, such as body weight, blood pressure and heart rate, as well as patient-reported symptoms, telemonitoring may support earlier recognition of clinical deterioration and allow timely therapeutic adjustments, potentially reducing the likelihood of acute decompensation and hospitalisation [2,3,5,7]. Importantly, its effectiveness appears to depend not only on data collection but also on structured clinical oversight and the capacity to respond promptly to abnormal findings. Technological approaches in this area have evolved considerably over the past two decades, ranging from simple telephone-based follow-up to more advanced digital platforms incorporating wearable devices, mobile applications, and implantable sensors capable of continuous physiological monitoring [7]. Increasingly, these systems are integrated into broader models of chronic disease management, facilitating closer collaboration between patients and healthcare professionals and enabling more individualised care strategies.

However, the clinical utility of telemonitoring in HF remains a topic of ongoing debate. Although several randomised controlled trials and meta-analyses have reported reductions in HF-related hospitalisations and, in some instances, mortality, other studies have not demonstrated clear or consistent clinical benefit [2,8-10]. These divergent findings indicate that telemonitoring should not be regarded as a single, uniform intervention but rather as a broad set of approaches encompassing heterogeneous technologies and models of care. Variability in outcomes may reflect differences in patient characteristics, disease severity, follow-up duration and selected clinical endpoints, as well as marked heterogeneity in monitoring intensity and clinical response pathways. Findings from published studies suggest that programmes incorporating structured clinical oversight and proactive therapeutic adjustment may achieve more favourable outcomes than those that rely primarily on passive data collection [2]. Thus, the effectiveness of telemonitoring appears to depend not only on the technology used but also on the extent to which it is integrated into a coordinated HF management strategy.

This review provides an overview of contemporary telemonitoring approaches in HF, summarising current knowledge across non-invasive, invasive, and mobile-based systems. We outline the range of monitoring modalities, describe their reported effects on clinical outcomes such as hospitalisation and mortality, and discuss factors that may account for variability in study findings. Particular emphasis is placed on programme design, monitoring intensity, and clinical response processes, as these elements appear to influence overall effectiveness. The aim is to support a clearer understanding of how different telemonitoring strategies operate in practice and to highlight considerations relevant to their integration into routine HF care.

Methods

This study was conducted as a narrative review with the aim of summarising key themes, technological developments and clinical insights related to telemonitoring in heart failure. As the review was not designed as a systematic or scoping review, formal Preferred Reporting Items for Systematic Reviews and Meta-Analyses (PRISMA) methodology was not applied.

A literature search was performed in PubMed to identify influential and clinically relevant publications. The search covered studies published between 1 January 2009 and 30 June 2025, with the final search conducted on 30 June 2025. The following search string was used: (“heart failure” AND (“telemonitoring” OR “remote patient monitoring” OR “telemedicine” OR “mobile health” OR “digital health”)).

In keeping with the narrative focus of this review, PubMed was selected as the primary database because it provides broad, clinically oriented coverage of heart failure and digital health research. The aim of this review was concept-driven rather than exhaustive; therefore, additional databases such as Web of Science, Scopus or the Cochrane Library were not included, as the intention was to capture key themes, influential publications and representative clinical developments rather than to conduct a systematic literature sweep.

The search was restricted to English-language full-text articles. Commentaries, editorials, conference abstracts and studies focusing exclusively on tele-rehabilitation or non-clinical monitoring were excluded. No restrictions were applied regarding study design.

Titles and abstracts were screened by the authors to identify publications relevant to the scope of this narrative review. Full texts of potentially eligible articles were assessed, and disagreements were resolved through discussion. The initial search resulted in 55 records; after removal of duplicates, 34 articles remained, of which 21 were included in the final narrative synthesis.

Data extracted from the included studies focused on high-level characteristics relevant to conceptual synthesis, including study design, patient population, type of telemonitoring intervention (invasive or non-invasive), monitoring intensity, duration of follow-up and reported outcomes (hospitalisations, mortality and patient-reported outcomes).

Given the narrative nature of this review and the substantial heterogeneity among studies in terms of objectives, settings and intervention designs, no formal risk-of-bias assessment was performed. Instead, findings were synthesised qualitatively and organised thematically to highlight overarching patterns, consistencies and gaps within the existing evidence base. We acknowledge that limiting the search to a single database introduces potential selection bias, which is inherent to narrative reviews. Accordingly, the findings presented here aim to summarise predominant concepts and trends rather than to provide an exhaustive account of all available evidence.

## Review

Types of telemonitoring in HF

Telemonitoring encompasses a broad spectrum of technologies and care models used for the remote assessment of patients with HF. For the purposes of this review, telemonitoring approaches are grouped into non-invasive systems (e.g., weight and blood pressure monitoring, symptom reporting, mobile applications) and invasive or device-based systems that enable continuous physiological measurement via implanted sensors [2,7,10]. In this context, telemonitoring is defined as the structured, remote collection and clinical interpretation of patient-generated health data to support decision-making, rather than simple communication or follow-up. An overview of the main telemonitoring modalities and their characteristics is summarised in Table 1.

**Table 1:** Comparison of non-invasive and invasive telemonitoring approaches in heart failure BP, Blood pressure; HR, Heart rate; HF, Heart failure; SpO2, Saturation of peripheral oxygen. Data derived from Sousa et al. [5], Veenis et al. [7], Spethmann et al. [10].

Feature	Non-invasive Telemonitoring	Invasive Telemonitoring
Parameters monitored	Weight, BP, HR, SpO₂, patient-reported symptoms	Pulmonary artery pressure, intracardiac pressure, thoracic impedance, HR, rhythm, medication adherence
Data collection	Patient self-report, home devices, mobile apps	Implantable sensors, continuous automated collection
Clinical oversight	Multidisciplinary team, periodic review	Multidisciplinary team, real-time alerts, algorithm-guided intervention
Monitoring intensity	Intermittent, daily, or multiple times per week	Continuous, 24/7
Patient population	Broad, including mild/moderate HF	High-risk, post-discharge, advanced HF
Effectiveness	Reduces hospitalisation, mixed impact on mortality	Reduces hospitalisation and mortality, most effective with structured care
Limitations	Adherence, data accuracy, digital literacy	High cost, technological infrastructure, personnel training, reimbursement challenges

Non-invasive Telemonitoring

Non-invasive telemonitoring represents the most frequently used approach in remote HF management and includes a broad range of tools for collecting patient-generated health data. As summarised in Table 1, these systems typically monitor symptoms (e.g., dyspnoea, fatigue) and physiological parameters such as body weight, blood pressure, heart rate, or oxygen saturation, which are transmitted from the patient’s home environment [7-9,11].

Available models vary widely, from telephone-based follow-up to integrated digital platforms with automated alerts. In most programmes, transmitted data are reviewed by clinicians, often within multidisciplinary teams, who adjust treatment or initiate triage procedures when predefined thresholds are exceeded [8,9]. Many non-invasive programmes also incorporate educational components aimed at improving self-care and adherence [1,2,6,12].

More recent approaches increasingly rely on mobile health applications that provide real-time connectivity and interactive features to support patient engagement [13]. Although these solutions are scalable and relatively low-cost, their effectiveness is closely linked to patient adherence and the reliability of the submitted data. The heterogeneity of protocols, from basic weight monitoring to more complex, algorithm-supported models, likely contributes to the variability in clinical outcomes reported in the literature [2,7].

Single-parameter monitoring, particularly daily weight measurement, has shown limited sensitivity for detecting early decompensation. Consequently, many contemporary non-invasive programmes employ multiparametric monitoring, combining physiological measurements with patient-reported symptoms to improve early detection. However, the performance of such strategies remains dependent on consistent patient participation and accurate data entry, underscoring the need for structured education and ongoing clinical support.

Invasive Telemonitoring

Invasive telemonitoring involves the use of implantable devices capable of continuously recording physiological signals. These include cardiac implantable electronic devices (CIEDs), such as pacemakers and defibrillators, as well as dedicated sensors that measure intracardiac or pulmonary artery pressures [5,7,14]. Some systems also estimate thoracic impedance, which may offer early indications of fluid accumulation before overt symptoms develop.

As summarised in Table 1, invasive monitoring generates device-derived data streams that can be integrated into clinical platforms and reviewed by specialised HF teams. In some programmes, algorithm-based decision pathways are used to support treatment adjustments when predefined thresholds are exceeded [8,10]. These structured approaches aim to standardise interpretation and facilitate timely clinical response, although the extent of automation and the clinical roles involved vary considerably across programmes.

Findings from existing studies suggest that invasive monitoring may reduce HF-related hospitalisations in select patient groups, particularly when monitoring is combined with clearly defined clinical workflows and active therapeutic adjustment [10,11,15,16]. However, results across trials remain heterogeneous, reflecting differences in patient populations, device types, and the organisation of monitoring pathways. Mortality benefits have been inconsistent, with some meta-analyses reporting positive signals and others showing neutral effects.

Invasive strategies may be particularly useful during the early post-discharge phase, when the risk of clinical deterioration is high and more intensive follow-up is needed [6]. When incorporated into broader HF management frameworks, these programmes may also support patient engagement and adherence to guideline-directed therapy [1].

Despite their potential advantages, invasive telemonitoring systems require substantial resources, including technological infrastructure, trained personnel, and sustained organisational support [10]. Variations in healthcare delivery models and reimbursement policies may limit scalability [2], and further research is needed to clarify which patient groups derive the most benefit [5,14].

 Clinical effectiveness of telemonitoring

Telemonitoring's impact on HF management has been rigorously assessed across key metrics: mortality, hospitalisation rates, and quality of life [4,8,15,16].

Impact on Mortality

Findings from randomised controlled trials and meta-analyses examining mortality outcomes remain mixed. Several analyses have reported reductions in all-cause mortality among patients enrolled in telemonitoring programmes [2,4,8,17,18]. However, these effects are not consistent across studies and appear to depend strongly on the specific features of each intervention.

Survival benefits are more frequently observed in programmes with structured, high-intensity monitoring, typically involving frequent data transmission, clearly defined clinical response protocols and active involvement of specialised HF teams [14,18]. In contrast, interventions characterised by infrequent data review or delayed clinical action (“passive monitoring”) have shown limited or no effect on mortality [3,11].

Heterogeneity in patient populations (including variation between HFrEF and HFpEF), intervention duration, monitoring intensity, and the nature of clinical actions triggered by the data likely contributes to the divergent results reported in the literature [2,3,7].

Overall, while mortality reductions have been reported in selected contexts, telemonitoring cannot be considered uniformly beneficial. Programmes embedded within coordinated, multidisciplinary care pathways, particularly those targeting higher-risk patients, appear more likely to demonstrate favourable survival signals, but these findings are not universal [1,14].

Impact on Hospitalisations and Readmissions

Findings on hospitalisation outcomes are generally more favourable than those reported for mortality, although they remain heterogeneous across studies. Several systematic reviews and meta-analyses have described reductions in HF-related hospitalisations among patients enrolled in telemonitoring programmes, with some reporting modest decreases in all-cause admissions as well [3,4,8,15-17,19,20]. In certain high-risk populations, reductions in HF-related readmissions of approximately 20-35% have been reported, though these effects are not universal and vary according to programme structure and patient profile.

Proposed mechanisms underlying reduced hospitalisations include earlier recognition of haemodynamic congestion, detectable through trends such as gradual weight gain or changes in blood pressure, before the onset of overt clinical symptoms [4]. These early signals may create opportunities for timely outpatient interventions, including diuretic adjustments, which can mitigate progression to acute decompensation [13,14].

More favourable outcomes have been observed in programmes implemented during the early post-discharge phase, when the risk of recurrent deterioration is highest. Interventions characterised by frequent monitoring, clearly defined alert algorithms, and prompt clinical action tend to report greater reductions in readmission rates [3,6,7]. Conversely, low-intensity or passive monitoring without structured clinical response pathways has shown limited impact.

Overall, the variability in hospitalisation outcomes highlights the importance of how telemonitoring is operationalised. Programmes embedded within structured transitional care pathways and supported by proactive clinical management appear more likely to demonstrate benefit, although results remain inconsistent across the literature.

Quality of Life, Adherence, and Patient-Reported Outcomes (PROs)

Several studies have explored the effect of telemonitoring on PROs in HF, although findings remain heterogeneous. Some interventions have reported improvements in health-related quality of life (HRQoL), including reductions in symptom burden or psychological distress, while others have shown minimal or no change [1,12,13,18]. Variability in PRO results likely reflects differences in programme design, monitoring intensity, and the specific tools used to assess quality of life.

Telemonitoring may also support better self-care and medication adherence by providing structured feedback, reminders and educational content. Such features can increase the awareness of early signs of deterioration among patients and promote more timely self-management behaviours. However, adherence to telemonitoring programmes varies widely, and factors such as age, digital literacy, cognitive impairment, and perceived monitoring burden can substantially influence patient engagement [1].

While improvements in PROs have been reported in some contexts, these outcomes are not consistently observed, and the overall impact of telemonitoring on quality of life remains uncertain. Importantly, PRO effects appear to be stronger when telemonitoring is delivered as part of a structured, responsive care model with active clinical oversight. Overall, these findings suggest that the potential benefits of telemonitoring extend beyond traditional clinical endpoints, but remain highly dependent on patient engagement and programme design.

From a broader perspective, telemonitoring has the potential to influence several dimensions of HF management. Some studies report reductions in hospital admissions, particularly those related to early decompensation, when telemonitoring is embedded within structured care pathways and supported by proactive clinical decision-making. Its impact on all-cause mortality remains inconsistent; however, more favourable results tend to be observed in programmes characterised by intensive monitoring and active clinical oversight [2,8,9].

These observations suggest that the contribution of telemonitoring may extend beyond traditional clinical endpoints, with possible benefits for patient experience, engagement, and day-to-day disease management. Nevertheless, such effects are not universal and depend heavily on programme design, patient adherence, and the overall care framework within which telemonitoring is implemented.

The consistency of reported outcomes varied across studies. Conclusions were synthesised based on the concordance of findings in major meta-analyses and are presented in Table 2.

**Table 2:** Evidence map linking telemonitoring modalities to clinical outcomes PA, Pulmonary artery; CIED, Cardiac implantable electronic device; HFrEF, Heart failure with reduced ejection fraction; HF, Heart failure. Data were derived from Kwaah et al. [2], Polisena et al. [3], Pekmezaris et al. [4], Kimchi et al. [6], Veenis et al. [7], Parente et al. [8], Clarke et al. [12], Rebolledo del Toro et al. [13], Verses et al. [14], De Lathauwer et al. [15], Umeh et al. [17], Stevenson et al. [18].

Telemonitoring modality	Target outcome	Direction of effect	Key sources
Structured non-invasive (daily weight/symptoms + active clinical response)	Mortality	Possible decrease. Benefits reported mainly in structured, team-based programmes with rapid intervention pathways.	[2,14,18]
	HF Hospitalisations	More consistent decrease. Greatest effect seen in high-intensity, proactive programmes.	[3,8,15]
Passive/Low-intensity non-invasive (data collection without prompt response)	Mortality	Neutral/inconsistent. Most trials show no survival advantage compared with usual care.	[3]
	Readmissions	Neutral / small effect. Limited benefit without active clinical decision support.	[4,17]
Invasive telemonitoring (PA pressure sensors, CIED diagnostics)	Mortality	Mixed findings with some favourable signals. Benefits most often reported in selected HFrEF populations.	[14]
	HF hospitalisations	Reduction reported in several studies, particularly with PA pressure-guided care.	[7,10]
Post-discharge/Transitional monitoring (<30–90 days)	30-day Readmissions	Reduction reported, especially during the early “vulnerable phase,” though results vary by programme intensity.	[3,6,7]
All modalities	Quality of Life (QoL)	Improvements reported in some studies, but results remain heterogeneous; effects influenced by adherence and programme design.	[12,13]

Study diversity and heterogeneity of results

Although the evidence on telemonitoring in HF has expanded substantially, reported outcomes remain heterogeneous. Differences in intervention design, monitoring intensity, clinical response pathways, and patient populations contribute to the wide variation in results across studies [2,7-9].

Technological and Methodological Variance

Variation in telemonitoring outcomes reflects substantial technological and methodological diversity across interventions. Studies encompass a wide range of systems, from early telephone-based support and intermittent nurse follow-up to contemporary digital platforms integrating mobile applications, wearable sensors, cloud-based data environments and implantable haemodynamic monitors [2,7,10,11]. These differences affect the type, frequency and clinical relevance of the data collected, as well as the speed and structure of the corresponding clinical response.

Device-based systems such as cardiac implantable electronic devices (CIEDs) and pulmonary artery pressure sensors provide continuous physiological data and may enable earlier detection of deterioration, which has been associated with reductions in hospitalisations in several studies [5,7,14]. However, their use is limited by procedural invasiveness, cost and infrastructure requirements. Non-invasive approaches are generally more scalable and accessible but depend heavily on patient engagement, digital literacy and adherence to monitoring protocols [8,11]. Variability in patient behaviour, data completeness and programme design adds further methodological complexity and contributes to the heterogeneity observed across telemonitoring research.

Patient Selection and Protocol Standardisation

Variation in patient characteristics contributes substantially to the heterogeneity of telemonitoring outcomes. More favourable effects are often reported in higher-risk groups, such as patients with advanced HF, multiple comorbidities or recent hospitalisations, where the likelihood of clinical instability is greater [2]. In contrast, studies involving patients with milder disease frequently report neutral findings. The effectiveness of patient-led monitoring may also be influenced by factors such as digital literacy, social support and engagement of the patients with their disease and treatment [2,10,12].

Differences in monitoring protocols further complicate comparisons across studies. Telemonitoring programmes vary widely in reporting frequency, alert thresholds and the structure of the clinical response pathway [2]. Interventions with timely, well-defined response mechanisms are more likely to demonstrate benefit, whereas programmes characterised by delayed or unclear clinical action tend to show limited impact [10]. Lack of standardisation in these components remains a major source of variability in reported outcomes.

Phenotype-Specific Evidence Gaps

Despite the growing body of research on telemonitoring in HF, phenotype-specific evidence remains uneven, with most clinical studies conducted predominantly in HFrEF populations [7,15,16]. In contrast, evidence for HFpEF is limited, as few telemonitoring trials include sufficient numbers of HFpEF patients or provide stratified analyses to assess whether similar benefits apply to this group [7,16]. Differences in pathophysiology, comorbidity burden, and clinical trajectories further complicate extrapolation from HFrEF results. Future research should prioritise adequately powered, phenotype-specific evaluations to clarify the effectiveness and clinical role of telemonitoring in HFpEF.

Telemonitoring after hospital discharge

The transition from hospital to home represents a period of heightened vulnerability for patients with heart failure, marked by increased risks of early rehospitalisation and mortality. Telemonitoring has been explored as a means of extending clinical oversight during this phase and supporting continuity of care when clinical stability is still evolving [3,6].

Results from pilot studies and systematic reviews suggest that the timing of telemonitoring initiation may influence outcomes. Programmes introduced immediately after discharge and maintained throughout the early recovery period have reported reductions in readmission rates in several studies [3,6]. By facilitating frequent assessments of weight, blood pressure and symptoms, these systems may allow earlier recognition of fluid accumulation and other signs of deterioration before urgent hospital care is required [2].

However, technology alone does not appear sufficient. More favourable results are typically seen in interventions that combine remote physiological monitoring with structured clinical components, including patient education, predefined escalation pathways and timely review by clinicians experienced in HF management [15,18].

In contrast, programmes characterised by short monitoring durations, passive data collection without clinical feedback or limited integration with outpatient care pathways often report neutral findings [3,4,17,18]. These variations underscore the importance of programme design and implementation fidelity in determining effectiveness.

Overall, telemonitoring during the post-discharge period shows promise for reducing preventable readmissions, but its impact depends on the structure, intensity and responsiveness of the monitoring model used. Future research should focus on identifying the optimal duration of monitoring and determining which patient subgroups derive the greatest benefit from this form of intensified follow-up [2,6,7,18].

Technology in telemonitoring

Monitoring Devices and Measurement Systems

Technological developments in both non-invasive and invasive monitoring tools have broadened the range of options available for remote HF management. Non-invasive systems remain the most widely used due to their simplicity, safety and suitability for home-based assessment. These approaches typically rely on devices that measure body weight, blood pressure and oxygen saturation, often supplemented by patient-reported symptoms [8,11]. Their accessibility makes them appropriate for large-scale implementation, particularly in patients with stable or moderately symptomatic disease.

Invasive telemonitoring relies on implantable cardiac devices and haemodynamic sensors capable of providing continuous physiological data. These systems can detect early signs of deterioration in higher-risk populations, although evidence of their comparative advantage over non-invasive approaches remains mixed. Their broader use is limited by procedural risks, costs and infrastructure requirements, restricting deployment mainly to specialised centres [7].

Importantly, the effectiveness of any telemonitoring technology depends not only on the monitoring tools themselves but also on how transmitted data are interpreted and incorporated into clinical decision-making. Telemonitoring functions as a supportive element within HF management, and its impact is determined by the organisational and clinical context in which it is implemented [2,10,18].

Mobile Applications and Digital Platforms

Mobile applications and digital platforms are increasingly incorporated into HF telemonitoring programmes. These tools allow for frequent or real-time data transmission, automated alerts and two-way communication between patients and clinicians [13]. Many systems also provide educational content and medication reminders, which may support treatment adherence and self-management [1,2].

Findings from systematic reviews suggest that mobile-based telemonitoring can reduce HF-related healthcare utilisation and improve adherence in some settings, particularly when combined with structured clinical oversight [8]. Compared with telephone-based approaches, mobile applications may offer advantages in scalability and ease of implementation, although their impact varies across studies [13].

Patient engagement with these platforms depends on factors such as digital literacy, age, usability and the perceived burden of daily monitoring [1]. Features such as intuitive interface design, personalised feedback and reliable technical support may help maintain adherence and user satisfaction [1,13]. At the same time, challenges related to data privacy, interoperability and regulatory requirements remain important considerations for widespread adoption [10].

Barriers to telemonitoring implementation

Despite growing interest in telemonitoring for heart failure, several factors hinder its broader implementation. A major challenge is the substantial initial investment required for equipment, software, staff training and ongoing technical support. Although reductions in long-term healthcare utilisation are possible, the upfront financial burden, along with uncertainties regarding the optimal duration of monitoring, remains a barrier, particularly in resource-limited settings [7,20].

Another obstacle is the lack of standardised protocols. Considerable variation exists in monitored parameters, alert thresholds, data review frequency and clinical response pathways, which complicates programme implementation and contributes to heterogeneity in study outcomes [7,9,10]. The absence of widely accepted guidelines also limits scalability and comparability across systems.

Organisational and behavioural factors additionally influence adoption. Clinicians may be concerned about increased workload or integration with existing workflows, while patients may have reservations related to data privacy, technical complexity or the perceived burden of continuous monitoring [10]. Addressing these concerns through clear role definition, adequate training and transparent communication is essential for improving acceptance and quality of life [1].

Finally, disparities in access to technology and digital skills present significant challenges. Limited internet access, especially in rural or low-income regions and lower digital literacy among older adults can restrict participation in telemonitoring programmes [10]. Enhancing digital inclusion and providing tailored technical support will be crucial to ensuring equitable access and preventing a widening of existing health disparities.

Future perspectives and research directions in telemonitoring

Ongoing advances in digital health technologies continue to shape the development of telemonitoring for HF. Future research may focus on improving the accuracy and clinical relevance of monitoring systems, integrating telemonitoring into comprehensive care pathways, and tailoring interventions to individual patient needs and risk profiles [10,21].

Integration with Home-Based and Community Care

An emerging trend in HF management is the integration of telemonitoring into home- and community-based care pathways. Rather than functioning as a standalone intervention, telemonitoring is increasingly incorporated into coordinated care models involving home-care nurses, general practitioners and specialist HF teams [11,18]. Such collaboration allows remotely collected data to be interpreted within the context of routine clinical care, supporting more timely decision-making and individualised therapy adjustments.

Experience from national healthcare systems, including Germany’s recent telemonitoring initiatives, suggests that system-level integration may improve continuity of care, strengthen communication between providers and reduce fragmentation during transitions between hospital and home [10]. Embedding telemonitoring within existing organisational structures, such as primary care networks, chronic disease management programmes and reimbursement frameworks, may also promote long-term sustainability and more consistent real-world implementation.

Artificial Intelligence (AI) and Prediction of HF Decompensation

Emerging applications of AI and machine learning are increasingly explored as potential tools to enhance telemonitoring in HF. These approaches analyse large volumes of physiological signals, patient-reported data and device-derived metrics to identify subtle and multidimensional patterns that may precede clinical deterioration [1,10,21]. In contrast to traditional threshold-based alert systems, AI models may incorporate individual baseline variability and temporal trends, potentially reducing unnecessary alerts and improving the relevance of clinical notifications.

By supporting earlier risk identification, AI-enhanced telemonitoring could facilitate a shift from reactive to more proactive management strategies. Earlier recognition of instability may, in some contexts, help clinicians intervene before overt decompensation occurs, although current evidence remains preliminary and uneven across studies [9].

Successful integration of AI into telemonitoring systems will require rigorous external validation, transparency regarding model performance and limitations, and careful alignment with existing clinical workflows to ensure safety, usability and trustworthiness. Considerations related to data quality, algorithmic bias and generalisability across diverse patient populations remain key areas for future investigation.

Personalised and Technology-Driven Care Strategies

Personalisation is an emerging focus in the development of telemonitoring for HF. Newer systems are being designed to adjust monitoring frequency, alert thresholds and clinical response strategies based on individual patient characteristics, disease severity, comorbidity burden and patterns of previous clinical instability [1]. Advanced implantable sensors and other invasive monitoring tools may support this approach in selected high-risk populations by providing more detailed haemodynamic data to inform therapeutic decisions [7].

Despite these technological developments, further research is required to determine their long-term clinical impact, cost-effectiveness and applicability across diverse patient groups. Important considerations include data privacy, transparency of artificial intelligence-supported decision pathways and the ethical implications of increasingly automated monitoring processes [21]. Large-scale trials and robust real-world evaluations will be essential to establishing whether personalised, technology-driven strategies translate into measurable improvements in patient outcomes [5].

## Conclusions

Telemonitoring represents a promising adjunct to conventional HF management, particularly with regard to reducing HF-related hospitalisations in well-structured programmes that incorporate timely clinical responses and multidisciplinary involvement. However, its effectiveness varies widely across studies and depends on factors such as the monitoring modality, the degree of clinical integration, and patient characteristics, including disease severity and engagement. The substantial heterogeneity of reported outcomes indicates that telemonitoring should be viewed as a context-dependent approach rather than a uniform intervention. Important evidence gaps remain, particularly regarding standardised protocols, long-term outcomes and the applicability of telemonitoring in older, frail and multimorbid populations. Future research should prioritise rigorous programme standardisation, inclusion of broader patient groups and evaluation of long-term clinical, organisational and PROs to better define the role of telemonitoring in routine HF care.
